# Extended Result Reading Window in Lateral Flow Tests Detecting Exposure to *Onchocerca volvulus*: A New Technology to Improve Epidemiological Surveillance Tools

**DOI:** 10.1371/journal.pone.0069231

**Published:** 2013-07-23

**Authors:** Allison Golden, Cathy Steel, Lindsay Yokobe, Emily Jackson, Rebecca Barney, Joseph Kubofcik, Roger Peck, Thomas R. Unnasch, Thomas B. Nutman, Tala de los Santos, Gonzalo J. Domingo

**Affiliations:** 1 Diagnostics Group, PATH, Seattle, Washington, United States of America; 2 The Laboratory of Parasitic Diseases, National Institute of Allergy and Infectious Diseases, National Institutes of Health, Bethesda, Maryland, United States of America; 3 Department of Global Health, Global Health Infectious Disease Research Program, University of South Florida, Tampa, Florida, United States of America; New York University, United States of America

## Abstract

Onchocerciasis is a neglected tropical disease caused by infection with the parasite *Onchocerca volvulus* (Ov). An estimated 180 million people are at risk for Ov infection, and 37 million people are infected, mostly in Africa. A lateral flow-based assay to detect human IgG4 antibodies to the Ov-specific antigen Ov-16 was developed as a rapid tool to detect exposure to Ov. The test, when performed on 449 sera specimens from patients with microfiladermia and Ov-negative patients, has a sensitivity of 89.1% (95% confidence interval: 86.2%–92.0%), and specificity of 97% (95% confidence interval: 95.4%–98.6%). Because the intended use of the test is for surveillance, it is highly desirable to have a stable, long-lasting result. An extended read window is thus desirable for a high-volume, busy workflow and facilitates post-surveillance quality assurance. The main restriction on achieving an extended read window for this assay was the erythrocyte lysis that can alter the signal-to-noise ratio, especially in those with low IgG4 levels (weak positives). We describe a test housing that incorporates a user-independent feature driven by assay fluid and an expanding wick that detaches the blood separation membrane from the nitrocellulose used in the assay, but before hemolysis occurs. We demonstrated material functionality at extreme operational conditions (37°C, 80% relative humidity) and a read window of a minimum of 70 days. The fluid-driven assay device performs equally as well with whole blood as with plasma, as demonstrated with 100 spiked clinical specimens (with a correlation coefficient of 0.96). We show a novel, inexpensive, and simple approach to actuating the detachment of the blood separation membrane from the nitrocellulose test with no impact on the performance characteristics of the test.

## Introduction

Onchocerciasis, or “river blindness,” is a treatable neglected tropical disease caused by infection with the parasitic helminth *Onchocerca volvulus* (Ov). The disease affects approximately 37 million people in Africa and the Americas, with over 500,000 people visually impaired and 250,000 people blinded by the disease [1]. The donation of the antiparasitic medicine – ivermectin – by Merck has enabled the development of large mass drug administration programs to reduce the burden of the disease. Combined with vector control activities, the burden of onchocerciasis has been reduced to elimination levels in both the Americas and Africa [2]–[5]. Recent data suggest that mass drug administration programs alone may achieve elimination in most areas, although additional interventions may also be required under certain circumstances [6]–[9].

Current tests for the definitive diagnosis of infection with Ov involve identification of subcutaneous nodules or direct observation of the Ov microfilariae by skin snip and microscopy. Skin snips combined with microscopy are the gold standard, but are relatively insensitive when microfilarial (MF) skin densities are low. Polymerase chain reaction of the skin snips using the O-150 repeat sequence as the target provides significantly greater sensitivity [10], [11] but is not, at the moment, suitable for either surveillance or point of care. As areas get close to transmission interruption and elimination and the disease burden is no longer significant within a community, reduced acceptability by the community members of skin-snip testing and the inadequate sensitivity of such testing become significant problems.

An alternative approach to the identification of incident infections in communities having already undergone mass drug administration involves using antibody detection to Ov-specific antigens that are expressed by the larval stages (L3 and L4) of the parasite. Several Ov-specific antigens have been assessed in the past [12]–[14]. The most widely used and the one adopted as a tool for monitoring control and elimination of onchocerciasis in the Americas by the Onchocerciasis Elimination Program in the Americas is the Ov-16 antigen [15]. Currently this test is performed by enzyme immunoassay (EIA or ELISA plate format) that detects IgG4 to this antigen. IgG4 detection results in a more specific test in comparison to IgG detection, leading to less false positive results. This is critical for a test used in a low prevalence, elimination scenario. Previously, a lateral flow rapid diagnostic test (RDT) was developed and evaluated but never commercialized [16], [17]. Field studies of this rapid format card test for Ov-16 (AMRAD, Australia) in West Africa demonstrated that MF prevalence rates correlated with antibody prevalence rates (Spearman’s *r* = 0.815; *P*<0.038). In Côte d’Ivoire, the Ov-16 card test had 76%–81% sensitivity (80%–92% positive predictive value) in communities without a history of ivermectin for vector control measures [17].

PATH, in collaboration with the National Institute of Allergy and Infectious Diseases, has been developing a lateral flow test for technology transfer to a manufacturer and commercialization. One of the key design input features is the stability of the test results output such that a reading 20 minutes after the test is performed gives the same result as a reading 24 hours or even weeks later. This allows for quality assurance of tests performed in the field as well as alleviating a time constraint in a busy work flow that is typical for staff members performing large-scale epidemiological assessments.

The Ov-16 RDT, as with many point-of-care lateral flow serology tests, requires red blood cell separation. The need to separate serum from platelets, white blood cells, and/or red blood cells may vary depending on the application, but is generally for reasons of chemical or visual separation. Red blood cells and their lysed fragments can visually obscure the test and/or control lines, resulting in invalid or false results and can even lead to costly product recalls. One of the device parameters for the Ov-16 RDT determined in the user-centric design process is to have test results that are stable from 20 minutes to 30 days. To achieve this using a finger-stick whole blood sample, the device must effectively separate red blood cells from serum and prevent them and their lysed fragments from entering into the reaction zone. Currently available products for separating red blood cells from serum used in manufacturing lateral flow tests fail with increased sample volume or release lysed fragments over time.

Here we demonstrate a housing strategy that ensures the stability of test results well beyond 30 days. The technology involves an expandable material to separate the blood separation membrane from the nitrocellulose test strip in an Ov-16 RDT after the sera has entered the test but before hemolysis of the red blood cells begins to occur. We demonstrate the performance of an Ov-16 RDT incorporated with this technology on 100 clinical specimens and compare the performance against microfilaria status and Ov-16 IgG4 status as determined by ELISA.

## Materials and Methods

### Specimens

All samples used were acquired under a number of registered protocols that were approved by the Institutional Review Board of NIAID with the majority being collected under NCT00001230. Written informed consent was obtained from all subjects prior to collection of the samples, and all the subjects consented to having serum stored for later analysis. Samples were either of serum or plasma and had been stored at −80°C until use. A positive specimen pool was made by combining four larger-volume specimens positive for IgG4 antibodies to Ov-16 antibody. The Ov-16 assay, both on the lateral flow test and on the ELISA, was evaluated on a panel of 449 sera specimens of known MF status (see Table 1). From this panel, a subset of specimens were used to create a spiked whole blood specimen panel by adding sera in equal volume to washed and packed red blood cells. Packed red blood cells were generated by centrifugation of type O-negative whole blood (in EDTA) and removal of plasma, with subsequent washes using phosphate-buffered saline, pH 7.4. This whole blood panel was composed of 50 *Onchocerca volvulus* MF-positive sera and 50 *Onchocerca volvulus*-negative sera. The spiked samples were used fresh within 8 hours of preparation.

**Table 1:** Inventory of specimens used to evaluate the Ov-16 lateral flow test.

Confirmed *Onchocerca volvulus* microfilariapositive or *Onchocerca volvulus* negative	Origin	Number used insera evaluation	Number used in whole-blood evaluation
**Positive**	Ghana	60	25
**Positive**	Liberia	48	0
**Positive**	Guatemala, Ecuador	110	25
**Positive**	US travelers	30	0
**Total ** ***Onchocerciasis volvulus*** ** microfilaria positive**		248	50
**Negative**	Liberia	1	0
**Negative**	Mali	18	10
**Negative**	*Loa loa* positive (US travelers)	40	0
**Negative**	Strongyloides (US travelers)	40	0
**Negative**	Lymphatic filariasis (origin Cook Islands and India)	43	20
**Negative**	Guatemala, Ecuador	37	10
**Negative**	US blood bank	22	10
**Total ** ***Onchocerca volvulus*** ** negative**		201	50
**Total number of specimens**		449	100

### Recombinant Ov-16 Antigen

Recombinant Ov-16 glutathione-S-transferase fusion protein was prepared through expression of the protein from a pGEX vector in *E.coli* and purification on glutathione-agarose columns (Thermofisher) as has been described previously [16].

### OV-16 ELISA

Ov-16 antigen is adsorbed to Immulon 2HB plates in 1× PBS, pH 7.4, at 5 µg/ml concentration overnight at 4°C. A solution of 1× PBS +0.05% Tween 20 and 5% fetal bovine serum (PBST+5% FBS) is used as both the blocking solution and assay diluent. Samples were typically diluted 50 and 100 fold in the assay diluent along with known positive and negative control samples. Mouse anti-human IgG4 antibody (Hybridoma Reagent Laboratory) was diluted 1∶5000 and goat anti-mouse IgG antibody, conjugated to horseradish peroxidase (Southern Biotech), was diluted to 1∶10,000 in the assay diluent. A 3,3,5,5 tetramethyl benzidine, TMB, reagent (Thermo Scientific) was used as the detection substrate and the reaction was stopped using 1N hydrochloric acid after a 15 minute incubation time at room temperature. Results were read using a 96-well plate reader and read at 450 nm. Positivity of the samples was assigned when the absorbance of the sample was above a defined plate-specific cutoff value, the average absorbance of the background (assay diluent only) +3 times the standard deviation of all background wells of the plate (6 per plate).

### Ov-16 Lateral Flow Test

*Materials in lateral flow strip:* Lohmann diagnostic backing material was used in card format with the dimensions of 300×75 mm. Assay HiFlow Plus (Millipore) nitrocellulose membrane was used in standard 25-mm width. Conjugate pad material was used in a 22-mm width. Absorbent wick was cut to 27 mm strips and buffer pad was cut to 15 mm-wide strips. Compressed cellulose wick material was purchased from Industrial Commercial Supply and cut to 27 mm-wide strips prior to assembly with the other lateral flow strip materials. All materials were assembled with a minimum of 2 mm overlap between materials, in 300 mm × 75–80 mm card format. Lateral flow strips were cut from the cards to a size of 75–80 × 4 mm.

### Assay Components

An Imagene IsoFlow Reagent Dispenser was used to dispense (0.025 mg) Ov-16 antigen and control antibody (goat anti-mouse, Jackson ImmunoResearch) stripes onto the assay membrane and to spray anti-human IgG4 antibody (Hybridoma Reagent Laboratory) conjugated to 40 nm gold colloid onto the conjugate pad material. These materials were assembled on the diagnostics card backing with the absorbent wick. For standard lateral flow strips used either without housing or as comparison tests within housing, a standard cotton fiber absorbent wick material was used; for the assay-actuated lateral flow tests, compressed cellulose wick material was used.

### Lateral Flow Strip Housing for Whole Blood Testing

The housing top was prepared using a 6-mil acetate film (McMaster Carr) overlay with punched holes. Double-sided tape adhered a 6×8-mm blood separation membrane (either Cytosep 1663 or Vivid GR, from Pall) to the acetate overlay. Double-sided tape applied to the back of the lateral flow strip backing prior to cutting strips was used to affix the test strips to a larger piece of diagnostic backing material cut to match the size of the housing top. The housing top overlay material with adhered blood filter was then placed on top of the lateral flow strip with the blood filter in contact with the lateral flow strip membrane.

### Lateral Flow Test Procedure

Tests run with sera were run directly on the lateral flow strip with no blood cell separation filter. A quantity of 2.5 µl of plasma was pipetted onto the assay membrane and followed by 10 µl of Ov-16 running buffer applied to the same spot. Sample and buffer then were allowed to run into the test for 2 minutes. The test strip was then inserted into a flat-bottom well containing 125 µl of running buffer and the test ran by capillary action. The panel of 449 specimens was run in this manner. For the spiked blood specimen panel, 15-µl blood specimens were applied to the blood filter region of the test and allowed to absorb. The specimen was chased by applying Ov-16 running buffer (sodium borate-based, 0.2% Tween), 15 µl for Vivid GR filters, and 30 µl for Cytosep 1663 filters, to the blood filter to facilitate sample flow in through the filter. Sample and buffer then were allowed to run into the test for 2 minutes. A total of 125 µl of Ov-16 run buffer was then added to the buffer pad upstream of the conjugate pad, and timing of the test began. Tests were evaluated at 5-minute intervals up to 25 minutes and then in the dry state (24 hours). Clinical specimen tests were monitored at 20 minutes, 1 hour, 24 hours, 10 days, 20 days, 30 days, and 70 days past test beginning.

### Determining the Signal Strength as a Function of the Assay Sequence

Sera samples were applied to either the conjugate pad upstream of the dried conjugate (BC), directly onto the conjugate (OC), or on the nitrocellulose upstream of the test and control lines (NC). Resulting dry signal strengths were measured using Image J by average pixel intensity across the strip width.

### Measurement of Compressed Material Response to Environmental Humidity and Temperature

Two medical-grade cross-linked polyvinyl alcohol (PVA) sponge sheets (Merocel, Medtronic) were tested that had different densities and wet thicknesses, either 3 mm or 6 mm. Sheet material of compressed cellulose at 3/8″ was purchased from Industrial Commercial Supply; 1 cm^2^ pieces of materials were cut from the sheets, and pre-exposure thicknesses were measured with microcalipers. The materials were then placed in an environmental chamber and exposed to a programmed temperature and relative humidity for the specified amounts of time at which the thicknesses of the pieces were measured and recorded.

### Evaluation of Endpoint Test Failures due to Blood Leakage onto Membrane

Lateral flow tests in housings were prepared as described above, using either Vivid GR or Cytosep 1663 blood filters, paired with either a standard absorbent wick material or compressed cellulose. Both types of wick materials were sized to absorb in excess of the total volume of fluid in the test. Tests were run with varying volumes of blood sample on all types of tests. A minimum of 10 tests, up to 100 tests, were run for each type blood filter, wick material, and volume. Endpoint test failure was decided in cases where blood leakage was present on the nitrocellulose in the region of the test and control line. Since there was a large qualitative range of the amount of blood leakage observed on the membrane, any presence of blood was noted as failure, whether or not the test was readable. Endpoint test failure was determined for the dry state (24 hours) of the test.

## Results

### Impact of Assay Sequence on Performance

**Figure 1:**
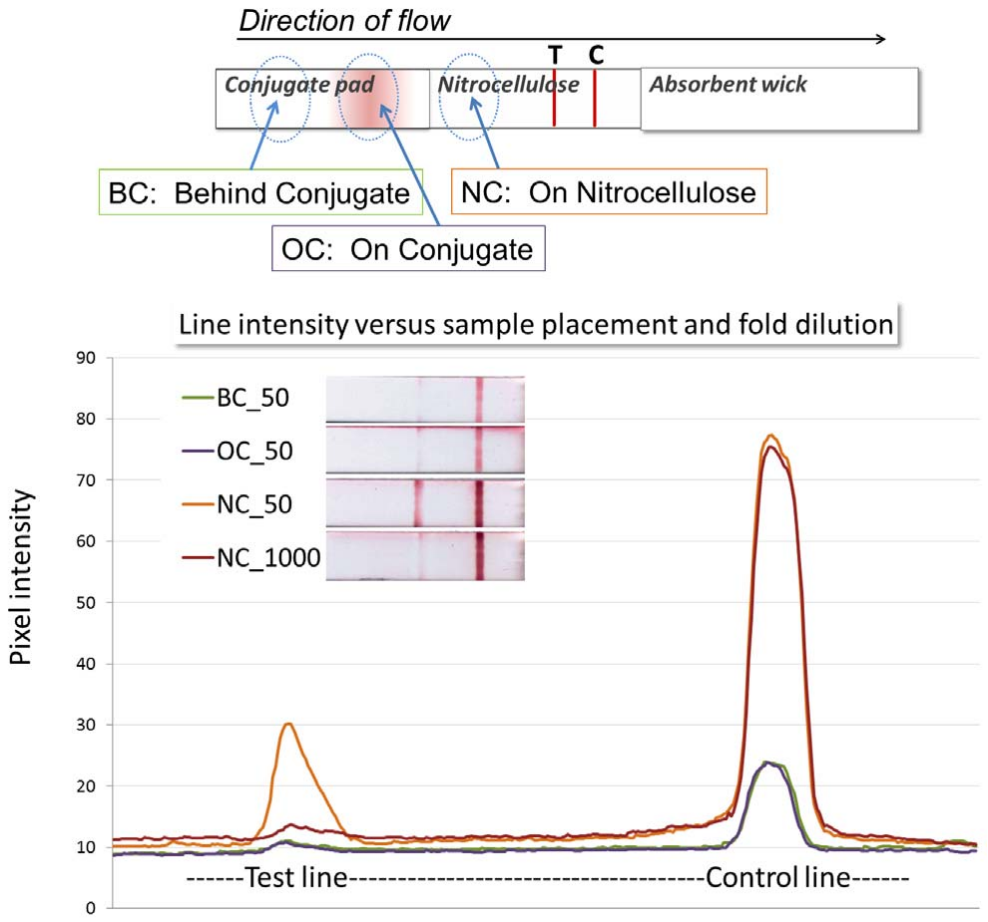
Signal strength of test and control line as a result of location of sample application. Diluted Ov-16-positive plasma samples were applied to either the conjugate pad upstream of the dried conjugate (BC), directly onto the conjugate (OC) or on the nitrocellulose upstream of the test and control lines (NC). Line scans show average pixel intensity across the width of the nitrocellulose strip.

The sensitivity of most lateral flow assays is strongly influenced by the mixing sequence of the different components of the analyte capture complex. The sequence can be changed by moving the location of the sample input with respect to the conjugate pad and the striped nitrocellulose. When three regions for sample input were tested with sera samples for overall signal development, only the sample input at the nitrocellulose generated specific true positives at the lowest limit of detection (1∶1000 dilution of positive pool), shown in Figure 1. We also observed that the control line intensity was also impacted by sample placement. This sequential addition was, therefore, an important feature of the test build.

### Assay-actuated Lateral Flow Test Housing to Detach Blood Membrane from Nitrocellulose

**Figure 2:**
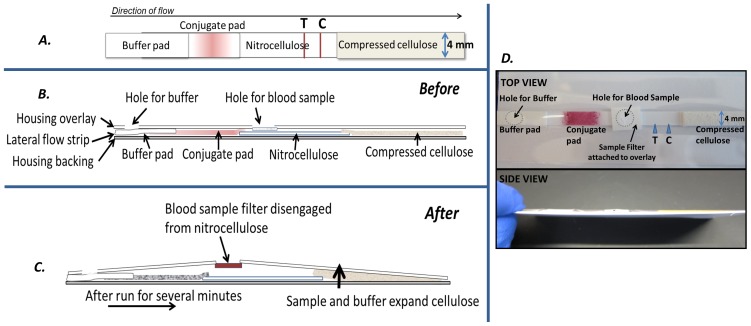
Schematic and pictures of Ov-16 soft cassette test. A) top view schematic of the basic Ov-16 lateral flow strip components, with compressed cellulose at the wick end noted (although Ahlstrom 243 absorbent material was also used when specified as standard wick material). B) Side view schematic of the test as assembled in the soft cassette housing, before fluid actuation. C) Side view schematic of a running test shown, with expansion of cellulose resulting in lift of overlay material and blood sample filter. D) Top and side views of the soft cassette housing, with test components labeled on top view.

One approach to address the common problem of hemolysis of the red blood cells in the blood separation membrane leaking into the nitrocellulose strip and causing background noise is to break contact between the blood separation membrane and the nitrocellulose after the sera has transferred on to the membrane. A solution was developed in which the blood membrane is detached from the nitrocellulose membrane by the assay fluid and an expanding wick pad without intervention by the test user. The blood separation filter is attached to the top cover of the lateral flow housing instead of being an integral part of the strip (Figure 2, B and D). The blood sample filter’s contact with the nitrocellulose strip can then be broken as the relative distance between the top cover and the strip increases. When the assay is run, the fluids of the assay rehydrate the wick, causing the wick to expand. The upward expansion of the rehydrating wick against the top layer of the lateral flow test housing carries the attached blood separation filter with it and thus disengages it from the surface of the nitrocellulose, preventing the leakage of blood into the test and preserving the clarity of signal (Figure 2C). A standard absorbent wick used in lateral flow tests expands only minimally (measured from several samples to be <0.2 mm) when exposed to fluid, requiring very narrow tolerances to achieve reproducible contact break. Compressed cellulose is capable of expanding over 10 times its dehydrated thickness, providing much broader tolerances and, as such, more robustness to test performance.

### Compressed Material Response to Environmental Humidity and Temperature

**Figure 3:**
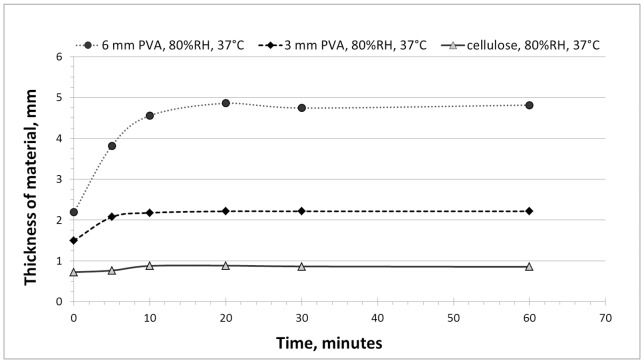
Thickness over time of three compressed hydrophilic materials. Sample thicknesses were measured after exposure to 37°C and 80% relative humidity over time.

In the device described, blood filter lift is actuated by expansion of the cellulose at the wicking end of the lateral flow strip. This expansion occurs rapidly when a solution comes into contact with the material and is a desirable feature of the material. However, it could be disadvantageous for the lack of contact to occur before the sample is added to the test, since it would reduce contact and transfer of plasma between the blood filter and the strip when the test is removed from its package. The possibility of humidity in the environment leading to premature expansion was a critical issue that needed to be evaluated before final choice of hydrophilic compressed wick material. The compressed cellulose and two PVA foam materials were evaluated by measuring the height of the material upon exposure to increasing temperature and humidity. All three expanding materials were relatively stable when exposed to moderate temperature and humidity (such as 30°C and 30% relative humidity). However, the two hydrophilic PVA foams (designated by their maximum thicknesses of 3 mm and 6 mm) expanded rapidly (in under 5 minutes) when exposed to high temperature and humidity, similar to those experienced in Africa where onchocerciasis control programs operate at 37°C and 80% relative humidity. In contrast, the compressed cellulose was relatively stable, increasing by only 0.1 mm under the most extreme conditions (Figure 3), despite its ability to expand to a thickness of over 6 mm when in contact with an aqueous solution.

### Impact of Whole Blood on the Performance of the Lateral Flow Test

**Figure 4:**
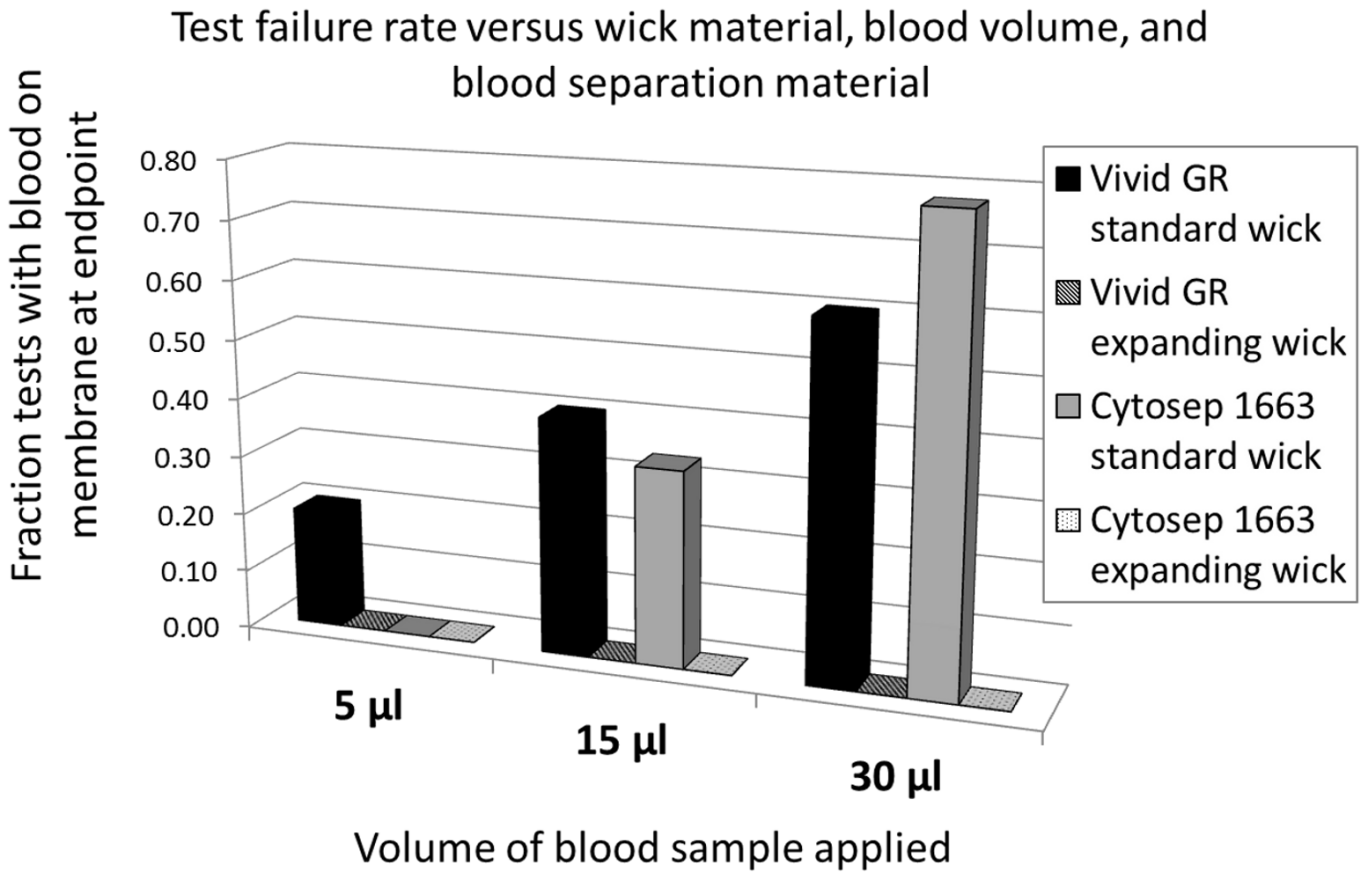
Fraction of tests that failed due to blood on membrane after dry. Tests that had blood on the membranes after the test was dry were counted as failed tests. This failure rate is shown as a function of the volume, blood filter, and wick material. A minimum of 10 tests were run for each condition shown.

**Figure 5:**
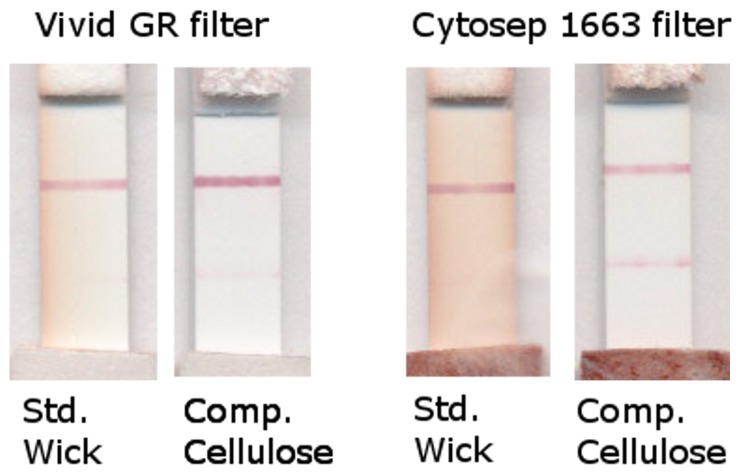
Example soft cassette tests run with standard wick (Ahlstrom 243) or compressed cellulose wick. All tests were run with positive-spiked blood samples. Vivid samples shown below were run with 5 µl of blood and Cytosep samples shown were run with 30 µl of blood.

The whole blood test required a separating membrane to transfer plasma to the assay membrane. Two types of blood filters were tested for use in this application. Cytosep 1663 is a thicker, more fibrous material. Vivid GR is a thin, smooth, asymmetric membrane. Both filter blood samples by size exclusion of the red blood cells, allowing the plasma to pass through into the test. However, red cells in contact with surfaces or air will rupture and hemolysis products will no longer be retained by the blood sample filter. For this reason, when the blood filter is placed directly on the optimal location for sample introduction (the nitrocellulose assay membrane), the resulting hemolysis is especially problematic given the proximity to the reading window. Any fluid remaining in contact with these hemolysis products will carry into the lateral flow strip. We examined the failure rate, as defined by presence of red blood cell components onto the membrane, as a function of both the blood filter type, the volume of the blood sample applied, and the type of absorbent wick, either standard or compressed cellulose. Test failures, although varied in both their onset and duration, were observed to emerge as early as 5 minutes into the test and also later than 30 minutes until the test became dry. Only endpoint failure (dry test) was considered for this analysis. A low volume (5 µl) produced no failures, but the blood sample volume is far under the capacity of the filter, resulting in less fluid contact between the filter and the nitrocellulose membrane and a drier sample filter which can draw fluid away from the strip, discouraging transfer of hemolysis products. While lower volume prevents failure, it also results in insufficient transfer of the plasma sample to the nitrocellulose membrane (data not shown), which could negatively impact the test results. The failure rate of tests using lateral flow strips with the standard absorbent wick increased sharply as the volume of blood sample increased, indicating that sample volume had some effect on the failure rate, but there were no observed test failures when the compressed cellulose wick was incorporated into the test strip (Figure 4) with either blood filter or with any volume of blood applied. This complete prevention of test failure was, therefore, due to removal of blood filter contact, as actuated by the expansion of the cellulose against the housing overlay and lift of the filter. Since even moderately larger volumes (15 µl or greater) increased the failure rate with both types of blood filters, it was clear that the standard wick was insufficient for the desired test performance. At low titers of target antibody where the signal-to-noise ratio on the strip is low, the added noise from the hemolysis products obscures the test line signal (Figure 5).

### Lateral Flow Test Performance with Clinical Sera Specimens

A blinded specimen panel of 449 specimens (described in Table 1) including confirmed Ov MF-positives and Ov-negatives was screened for IgG4 antibodies to Ov-16 by ELISA and the Ov-16 lateral flow test to establish the baseline performance of the lateral flow test itself. The specimens were from well-characterized subjects or patients seen by the Clinical Parasitology Section, Laboratory of Parasitic Diseases at the National Institutes of Health. The sera specimens were added upstream of the conjugate pad directly onto the nitrocellulose membrane, followed by buffer applied to the conjugate pad containing the gold conjugate. The lateral flow test and ELISA had sensitivities of 89.1% (95% CI: 84.4%–92.6%) and 84.3% (95% CI: 79.0%–88.4%) and specificities of 97% (95% CI: 93.3%–98.8%) and 94% (95% CI: 89.6%–96.7%), respectively, when compared to the Ov status (Table 2). The lateral flow test run with sera had a sensitivity and specificity against the ELISA of 96.4% and 93.9%.

**Table 2:** Performance of the Ov-16 lateral flow test and ELISA on a panel of sera samples against *Onchocerca volvulus* (Ov) status and Ov-16 enzyme immunoassay (ELISA).

		Ov-16 ELISA		Ov-16 lateral flow	
		*Positive*	*Negative*	*Positive*	*Negative*
**Ov status**	*Positive*	209	39	221	27
	*Negative*	12	189	6	195
**Ov-16 lateral flow**	*Positive*	213	14	–	–
	*Negative*	8	214	–	–

### Performance of the Assay-actuated Lateral Flow Test with Spiked Whole-blood Specimens

A blinded panel of 100 frozen sera specimens was selected from the larger panel of 449 specimens (Table 1). These were spiked into washed and packed O-negative red blood cells. With this subset of specimens, the assay-actuated lateral flow test performed similarly well with whole-blood specimens as the standard lateral flow test and ELISA did with sera specimens (Table 3). The assay-actuated lateral flow test using spiked blood samples had a sensitivity of 96.0% (95% CI: 92.2%–99.8%) and specificity of 96% (95% CI: 92.2%–99.8%) with respect to Ov status. The correlation coefficient between the assay-actuated lateral flow test with whole blood and lateral flow test run with sera samples was 0.96, showing that in addition to similar performance values, the vast majority of test results were in agreement. In this set of 100 tests performed by an independent user no mechanical or fluidic failures were observed.

**Table 3:** Performance of assay-actuated lateral flow test on spiked whole-blood samples compared to ELISA and the lateral flow test with sera.

Sample source	Number ofspecimens	ELISA withsera-positiveresults	Lateral flow testwith sera-positiveresults	Assay-actuated lateralflow test with wholeblood-positive results
**Confirmed ** ***O.volvulus*** ** microfilaria positives**				
**Guatemala, Ecuador**	25	24	25	24
**Africa, Ghana**	25	23	24	24
**Total positive results**	50	47	49	48
**Total negative results**	0	3	1	2
**Confirmed ** ***O.volvulus*** ** negatives**				
**Guatemala, Ecuador**	10	1	1	1
**Lymphatic filariasis** **(origin Cook Islands and India)**	20	0	0	0
**Africa, Mali**	10	0	0	1
**US blood bank**	10	1	0	0
**Total positive results**	0	2	1	2
**Total negative results**	50	48	49	48
**Sensitivity (95% CI)**		**94%** (89.3–98.7)	**98%** (95.3–100.7)	**96%** (92.2–99.8)
**Specificity (95% CI)**		**96%** (92.2–99.8)	**98%** (95.3–100.7)	**96%** (92.2–99.8)

Sensitivity and specificity values are calculated against microfilaria status. The 95% confidence intervals are indicated in parentheses for the sensitivities and specificities and were calculated using the normal approximation interval.

### Read Stability

The assay-actuated lateral flow tests performed on the 100 blinded spiked blood specimens were read at a number of different time points to determine if an optimum read window was present or if the test merely needed to be read following a set time period, such as 20 minutes. The used tests were stored at room temperature and humidity. Eight separate reads were taken. Tests were qualitatively called to be negative, very weak positive, weak positive, and positive. Tests were read at 20 minutes, 1 hour, 24 hours, 10 days, 20 days, 30 days, and 70 days. While individual readers also changed throughout time (which is the most likely scenario in the end-use case), the tests were also read by two readers at some time points to look at inter-reader variability as well. Three test reads had calls which would result in an equivocal result. These tests fluctuated between very weak positive and negative calls and did not appear to change with respect to time but only with respect to the reader, with different calls made for two of the tests on the same day by two different readers. For test concordance over time and throughout different readers, positive calls of any intensity were considered concordant, and there appeared to be no change in intensity of the positive signal over time. Final test results were called within 20 minutes in 99% of the tests. Only one test was recorded to change from negative result to weak positive between 20 minutes and 1 hour. No tests changed in result from 1 hour to overnight. Shown in Table 4 are the estimated sensitivities and specificities calculated from test reads at a given time. It is important to note that a single test difference accounts for a calculated 2% difference in either sensitivity or specificity. For all tests, the reads were well within the range for a 95% confidence interval, and any fluctuations in test reads are not numerous enough to be statistically significant. Outside of this particular study panel, test results generated from stability studies on the lateral flow strip have suggested that the read window extends beyond one year (data not shown).

**Table 4:** Sensitivity and specificity of the lateral flow test at different read times.

	*20 min.*	*1 hour*	*24 hours*	*Day 10*	*Day 20*	*Day 30^1^*	*Day 30^2^*	*Day 70^1^*	*Day 70^2^*
**Sensitivity**	0.94	0.96	0.96	0.96	0.96	0.96	0.96	0.98	0.96
**Specificity**	0.96	0.96	0.96	0.98	0.94	0.96	0.94	0.94	0.94
**Correlation coefficient**	–	0.98*	0.98*	0.98	0.98	0.96	0.98	0.96	0.98

Four different people read the test results over these time points. At days 30 and 70 the tests were read by two readers indicated by superscripts 1 and 2. For this sample size (n = 100), a change of 0.02 in sensitivity or specificity represents a discordance in 1 test result. For test development results throughout drying, the correlation coefficients for 1 hour and 24 hours were calculated relative to the 20 minutes test results(*). The remaining time points give the correlation coefficients calculated relative to the 24-hour dry reads.

## Discussion and Conclusions

A lateral flow test to detect human IgG4 antibodies to the Ov antigen Ov-16 was developed. The Ov-16 lateral flow test, when performed on a panel of 449 specimens including *Loa loa* and lymphatic filariasis specimens, had a sensitivity of 89.1% (95% CI: 86.2%–92.0%) and specificity of 97% (95% CI: 95.4%–98.6%). When compared to an ELISA test designed to detect IgG4 antibodies to the same Ov-16 antigen, the sensitivity and specificity were 96.4% and 93.9%, respectively. Discordance between these two tests only occurred with weak positive specimens where the cutoff of the ELISA and the signal-to-noise ratio in the lateral flow test were the determinants of concordance or discordance. The assay-actuated Ov-16 lateral flow test was evaluated for performance on a panel of 100 blood-spiked specimens, 50 of which were spiked with sera from Ov patients that were microfilaria positive, and 50 of which were spiked with sera from patients that were negative for Ov. The performance of the test was statistically identical to that of the ELISA and the lateral flow test ran with sera samples.

The sensitivity is similar to that reported for a previously developed (but not commercialized) Ov-16 RDT [16], [17]. Some of the false-positive results contributing to the specificity below 100% arose from patients from endemic countries, Ecuador and Mali, and from samples with limited data on patient histories. One *Loa loa*-positive sample that resulted in a false positive for Ov-16 was later reported to be from a patient suspected of having onchocerciasis, and one negative was from a field study worker who could also have had exposure. No other *Loa loa*-positive sample or any of the lymphatic filariasis-positive specimens gave false positives in this sample set. The intended use of the Ov-16 test is to detect exposure to Ov infection in a population that is in post-elimination stage of the disease, primarily in remote areas of Africa. Typically, this will be performed by surveillance teams sampling children under ten years old and in communities that have undergone years of mass drug administration and should no longer be exposed to the parasite. While specificity of the Ov-16 test is high, the use of a test with anything less than 100% specificity in confirmation of elimination will be challenging nonetheless, given that the true positivity rate could approach 0% [8]. However, in such end-use cases, a high specificity of 97% would allow identification of over 98% of the true negatives during surveillance of communities; only a small percentage would have to be followed up by a confirmatory test should they show positive.

The output for the assay-actuated Ov-16 test is stable to at least 70 days after performing the test, as read by multiple readers. A highly desirable feature for a diagnostics test is a stable test output whereby the test result is the same whether it is read immediately after the test is performed or several weeks after it was performed. If test results are not persistent, a limited read window is required. This limitation results in requiring an additional timing step for the individuals running the tests, which increases the risk for inaccurate reads when taken outside of the window of time, requiring retests in the case of missed windows. Extended test output stability allows programs to assure quality or confirm the data collected during surveillance.

A large number of RDTs require limited end-read windows in their product inserts, suggesting instability in the test result. Optimal immunoassay results arise from specifically increasing the relative signal-to-noise ratio. Complex samples such as blood will often contribute to the noise in a test system. Ideally, the analyte can be separated from the sample to reduce the noise in the test result. Lateral flow tests have a small fluid balance with which to perform separation, hydrate reagents, and wash away background and nonspecific interactions. It is common in many gold standard immunoassay techniques, such as dot blots and ELISA, to add sample and detection reagents in a stepwise fashion with large volume washes in between to ensure signal specificity and noise reduction. For the lateral flow test, stepwise and separated sample and buffer additions impact the housing of the test significantly since the sample port must be placed downstream of the conjugate in order for the sample to react with the test line prior to the detection conjugate. In the case of this Ov-16 lateral flow test, the signal-to-noise ratio is optimal if the specimen is allowed to bind to the antigen prior to exposure to the anti-IgG4 gold conjugate. The signal is stable over long periods of time when using plasma or sera only. However, when using whole blood, even with sample filtration, the signal may be obfuscated by eventual hemolysis of the red blood cells in the blood separation membrane and leakage into the assay membrane. This is particularly relevant in this case due to the fact that the specimen needs to be applied upstream to the conjugate pad directly onto the nitrocellulose for optimal sensitivity. The contact of the blood filter with the test membrane limits the read window and test robustness and it also increases dependence on changes in the volume of blood in the filter, the type of filter, and other factors which were not explored in these test runs including damage to the filter, temperature and humidity changes, or buffer volumes outside of the protocol volume. While removal of the blood filter can be achieved by adding a timed step in the test protocol at which the user mechanically removes the blood separation membrane after a short period of time, it is done at the expense of adding significant complexity and risk of user error to the assay procedure. To address this issue with minimal impact on the test procedure as experienced by the end-user, we designed a novel lateral flow test housing in which the blood separation membrane is detached from the nitrocellulose membrane after the plasma has entered the nitrocellulose by the fluids of the assay and an expanding wick material. We demonstrated that the material responsible for the actuation, the compressed cellulose, is robust to typical environmental conditions under which it will be operationally exposed in Africa (37°C and 80% relative humidity).

We demonstrated the performance of a user-independent way of detaching the blood separation membrane from the lateral flow test, with the timing consistent with optimal test performance while achieving the highly desirable product feature of an indefinite read window even for weak positives. The elimination of a user step to perform separation is important in reducing user failures. The extended read window is a critical product feature for a diagnostic test that will be used in remote areas in a surveillance context where many people are sampled in a short period of time. The technology is low cost, simple, and robust to high operational temperatures and humidity. The next step is to align this novel approach with manufacturing processes.
